# Vascular Dysfunction among Malaysian Men with Increased BMI: An Indication of Synergistic Effect of Free Testosterone and Inflammation

**DOI:** 10.3390/medicina55090575

**Published:** 2019-09-08

**Authors:** Amilia Aminuddin, Norizam Salamt, Ahmad Faiz Ahmad Fuad, Kok-Yong Chin, Azizah Ugusman, Ima Nirwana Soelaiman, Wan Zurinah Wan Ngah

**Affiliations:** 1 Department of Physiology, Universiti Kebangsaan Malaysia Medical Center, 56000 Cheras, Kuala Lumpur, Malaysia (N.S.) (A.F.A.F.) (A.U.); 2 Department of Pharmacology, Universiti Kebangsaan Malaysia Medical Center, 56000 Cheras, Kuala Lumpur, Malaysia (K.-Y.C.) (I.N.S.); 3 Department of Biochemistry, Universiti Kebangsaan Malaysia Medical Center, 56000 Cheras, Kuala Lumpur, Malaysia

**Keywords:** obesity, testosterone, pulse wave velocity, augmentation index, inflammation

## Abstract

*Background and objectives:* Obesity is associated with poor vascular function and may lead to future cardiovascular disease (CVD). Obesity is also related to increased inflammation and a low testosterone level. This study was conducted to determine the relationship between inflammation, testosterone level, and vascular function among subjects with an increased body mass index (BMI) and to determine whether both low testosterone and high inflammation have synergistic effects towards vascular dysfunction. *Materials and Methods:* A total of 303 men aged 40–80 years were recruited from Klang Valley, Malaysia. Their height, weight, blood pressure (BP), lipid, blood glucose level, total testosterone (TT), free testosterone (FT), and C-reactive protein (CRP) were measured. The carotid femoral pulse wave velocity (PWV_CF_) and augmentation index (AI) were also recorded as markers of vascular function. *Results:* The mean age of all the subjects was 54.46 ± 9.77 years. Subjects were divided into a low/normal body mass index (BMI) group (BMI < 25 kg/m^2^; NG, n = 154) and high BMI group (BMI ≥ 25 kg/m^2^; OG, n = 149). The mean BMI for NG was 22.20 ± 1.94 kg/m^2^ while for OG was 28.87 ± 3.24 kg/m^2^ (*p* < 0.01). The level of TT (OG = 21.13 ± 6.44 versus NG = 16.18 ± 6.16 nmol/L, *p* < 0.01) and FT (OG = 0.34 ± 0.12 versus NG = 0.39 ± 0.11 nmol/L, *p* < 0.01) were reduced while the level of CRP [OG = 1.05 (2.80) versus NG = 0.50 (1.50) mmol/L, *p* = 0.01] was increased in OG compared to NG. PWV_CF_ (OG = 8.55 ± 1.34 versus NG = 8.52 ± 1.42 m/s, *p* = 0.02) and AI (OG = 16.91% ± 6.00% versus 15.88% ± 5.58%, *p* < 0.01) were significantly increased in OG after adjustment for other CVD risk factors. The subjects that had both a low FT and an increased CRP had higher AI when compared to those with a high CRP and high FT (*p* < 0.01). *Conclusions:* The increased BMI was associated with vascular dysfunction, mediated by a low testosterone level and increased inflammation. Furthermore, having both conditions concurrently lead to higher vascular dysfunction. Weight loss, testosterone supplementation, and the anti-inflammatory agent may be beneficial for men to prevent vascular dysfunction.

## 1. Introduction

The prevalence of obesity is increasing rapidly in the developing world [1]. In Malaysia, the prevalence of overweight leapt from 17% in 1996 to 30% in 2015, whereas the prevalence of obesity escalated from 4% to 18% within the same period, which was a 4.5-fold increase [2,3]. Obesity is associated with poor vascular function, which potentially leads to cardiovascular disease (CVD) [4]. The vascular function can be accessed via the carotid femoral pulse wave velocity (PWV_CF_), which reflects aortic stiffness, and augmentation index (AI), which reflects the speed of pressure wave that travels from the aorta to the periphery as ventricles contract each cycle [5]. This pressure wave is reflected back to the heart and augments the pressure in the heart, which translates as the AI [5]. Both markers have been found to be associated with future CVD morbidity and mortality [6,7].

On the other hand, obesity is associated with a low testosterone level [8,9]. However, the relationship is complex and bidirectional. In obese individuals, the increased aromatase activity from fat mass leads to suppression of hypothalamic-pituitary-testis pathway, thereby reducing the production of testosterone from the Leydig cell in the testis [10,11]. Conversely, a low testosterone level promotes the formation of visceral fat, thus forming a vicious cycle that leads to more reduction in the testosterone level [12]. The increased leptin level among obese subjects also exerts a negative impact on the luteinising hormone (LH)/human chorionic gonadotrophin (hCG)-stimulated testicular androgen production [13]. The low testosterone level may lead to vascular damage by increasing the stiffness of the aorta and wave reflection [14,15].

The adipose tissue releases a variety of pro-inflammatory and anti-inflammatory mediators [16]. With the adipose hyperplasia and obesity, the blood supply to the adipocytes may be reduced, causing hypoxia [17]. Hypoxia triggers necrosis and macrophage infiltration into the adipose tissue and stimulates the inflammation cascade. Among the inflammatory mediators released are the tumour necrosis factor-alpha (TNF-α) and interleukin-6 (IL-6) [18]. IL-6 further stimulates the secretion of the C-reactive protein (CRP) from the liver, which promotes inflammation in the body [19]. This inflammatory mediator may reduce the nitric oxide (NO) availability in the blood vessel, causing endothelial and vascular dysfunction [20].

Limited human studies were done on the relationship between testosterone and inflammation with PWV_CF_ and AI. The presence of both conditions may worsen the vascular damage. Thus, this study aimed to examine the interrelation between the vascular function, inflammation, and testosterone level in overweight and obese subjects and to assess whether the presence of low testosterone and increased inflammation had synergistic effects on vascular dysfunction. The information generated from this study will be beneficial in identifying the risk factors of vascular dysfunction and potential areas of therapeutic for CVD diseases.

## 2. Materials and Methods

This study was part of the Malaysian Ageing Men Study [21]. The main study is a cross-sectional study determining the effects of ageing towards various aspects of health, such as cardiovascular health, bone health, and hormone level among men aged between 40 to 80 years, residing in Klang Valley, Malaysia. The protocol of this study was reviewed and approved by the Ethics Committee of Universiti Kebangsaan Malaysia (approval code: UKM-AP-TKP-09-2009).

### 2.1. Subjects Recruitment

Subjects were recruited using a purposive sampling technique from Klang Valley, Malaysia. Information on subject recruitment, including inclusion and exclusion criteria, was distributed by flyers, brochures, and advertisements on major newspapers and radio broadcasts. Health screening and data collection were conducted in selected community centres and religious places around Klang Valley. The inclusion criteria included men aged 40 to 80 years with no history of major cardiovascular events or other chronic debilitating illnesses. Those with cardiovascular risk factors, such as diabetes mellitus, hypertension, and dyslipidaemia were included. Subjects receiving sex hormones therapy, suffering from cancers or undergoing cancer therapy, had a fracture or underwent a major surgery six months prior to the screening date were excluded. Subjects were briefed on the details of this study and provided informed consent before enrollment.

The socio-demography and lifestyle details of the subjects were obtained using a questionnaire. Age was based on the birthday record on the identification card and ethnicity was self-declared. A smoker was identified as an individual who was smoking daily at the time of the study [22]. Medical history taking was performed by qualified physicians. Diabetes mellitus (DM) was defined by a fasting blood glucose (FBG) level of >6.1 mmol/L or undergoing the DM treatment [23]. Hypertension (HPT) was defined by a systolic or diastolic blood pressure ≥ 140 or 90 mmHg or those undergoing the hypertensive treatment [24]. Dyslipidemia was defined by a total cholesterol (TC) level > 6.2 mmol/l, or a low-density lipoprotein (LDL) level > 4.2 mmol/l, or high-density lipoprotein (HDL) < 1.04 mmol/l, or triglyceride (TG) > 1.7 mmol/L [25].

### 2.2. Anthropometry Measurement

A stadiometer and digital scale were used to measure the height and weight, respectively (SECA, Hamburg, Germany). The body mass index (BMI) was obtained by using the formula weight/height^2^ (kg/m^2^). The high BMI was defined as a BMI > 25 kg/m^2^, which covered both overweight and obese subjects [26]. Their waist circumference (WC) was recorded once by using a measuring tape to the nearest 1 cm at midway between the lower rib margin and the superior border of the iliac crest at the end of a normal expiration in the standing position [27]. Subjects were divided into subjects with a low/normal BMI (NG) and a high BMI (OG).

### 2.3. Measurement of Pulse Wave Velocity and Augmentation Index

The PWV_CF_ was measured by the Vicorder^®^ (SMT medical, Wuerzburg, Germany). The procedure was elaborated in a previous study [27]. Briefly, subjects had to lay supine with their heads supported by a pillow. Two recording cuffs were used, one cuff was positioned on the right thigh and another one was around the neck. The recording started as both cuffs were inflated to 65 mmHg. The delay between the two recorded pulse waves was measured by the device to get the transit time (tt). By using a measuring tape, the distance between the suprasternal notch to the mid-thigh cuff was measured and known as the distance of the pressure wave travelled (DPW). The software then computed the PWV_CF_ by dividing the DPW with the tt (m/s).

The cuff was also placed on the right arm to record the brachial BP pressure and waveform. The device then estimated the aortic pressure waveform using a brachial to-aortic mathematical transfer function as mentioned in the previous study [27]. The aortic pressure waveform was used to calculate the AI using the formula [(second systolic peak-first systolic peak)/pulse pressure × 100] [28]. The brachial BP was recorded once for every subject.

### 2.4. Measurement of Blood Parameters

Blood samples were taken in the morning after several hours of fasting. The blood was analysed by an accredited commercial laboratory (Gribbles Pathology, Petaling Jaya, Malaysia) for the measurement of the total testosterone, albumin, total cholesterol (TC), high density lipoprotein (HDL), low density lipoprotein (LDL), triglyceride (TG), and high-sensitivity CRP (hs-CRP) by using the automated analyser. The details of the measurement had been illustrated previously [27,29]. The free testosterone level was calculated using Sodergard’s formula [30]. The ACCUCHEK portable glucometer (Roche, Basel, Switzerland) was used to measure the fasting blood glucose (FBG) level by the glucose oxidase method.

### 2.5. Statistical Analysis

The normality of the data was examined using the Kolmogorov–Smirnov test. A comparison of basic characteristics between the NG and OG subjects was performed using the univariate analysis with adjustment for potential confounders. The associations between the total testosterone (TT), calculated serum free testosterone (FT) and CVD risk factors were determined using the bivariate Person’s correlation. The associations between the hs-CRP and other factors were determined by Spearman’s correlation since hs-CRP was not normally distributed. Differences between categorical parameters were determined using the Chi-square test. To determine the synergistic effects of the FT and hs-CRP towards the vascular function, the subjects were grouped into those with a high FT and low hs-CRP (G1), high FT and high hs-CRP (G2), low FT and low CRP (G3), and low FT and high CRP (G4), and their levels of PWV and AI were compared by using the one-way analysis of variance followed by Tukey’s pairwise comparison test. The cut-off point of the 50th percentile was used for the determination of the high and low level of FT and hs-CRP. The statistical significance was set at *p* < 0.05. All of the analysis was performed using the Statistical Package for Social Sciences (SPSS) version 16 (SPSS Inc., Chicago, IL, USA).

## 3. Results

Table 1 showed the biophysical and biochemical profiles of the subjects. 62.9% of the subjects were Chinese and 37.1% were Malays. The OG had a significantly higher BMI, BP, FBG, TG, and lower HDL, TT, and FT when compared to NG (*p* < 0.01 for all). OG also had a significantly higher AI and PWV_CF_ after adjustment for confounders, such as height, race, and HR, and other CVD risk factors (BP, TC, FBG, and smoking status). Adjustment was made for other CVD risk factors to highlight the influence of BMI alone on vascular functions. Dyslipidaemia was the most prevalent risk factor among them.

The correlation analysis revealed a significant negative association between free testosterone (FT) and total testosterone (TT) with major risk factors of CVD, such as age, WC, BMI, SBP, and hs-CRP (Table 2). For FT, an additional significant negative association was observed with AI (Table 2). The inflammatory marker, hs-CRP, was associated with PWV_CF_ (Spearman’s rho = 0.11, *p* < 0.05) and AI (Spearman’s rho = 0.14, *p* = 0.01).

Table 3 showed the subjects’ characteristics and vascular functions according to the groups of the high and low level of FT and hs-CRP. Grouping was based on the FT and not the TT since AI correlated with only FT. There was a significant difference for AI between the groups and post hoc analysis, which revealed that G4 had a higher AI compared to G1 and G2. This remained significant after adjustment for the age, HR, height, DBP, and smoker status.

## 4. Discussion

In this study, we aimed to identify the associations between testosterone, vascular functions, and inflammation among male subjects with a high BMI in Malaysia. We found that they had increased PWV_CF_ and AI compared to the subjects with a low/normal BMI. The hs-CRP was higher and the testosterone level was lower among the OG subjects compared to the NG subjects.

Abdominal obesity was a CVD risk factor in the NG group because their waist circumference was more than 90 cm [31]. Several studies had suggested a lower cut off point for normal BMI for Asians (BMI ≤ 23), since data showed that at a similar age and body fat, Asians had lower BMI [32,33]. In this study, 25 was used as the cut-off point for overweight because by lowering the BMI of the subjects to 23, we would have a much smaller sample size for NG and thus, further analysis could not be conducted.

### 4.1. Obesity and Vascular Function

The increased BMI was associated with decreased vascular functions [4]. We found that subjects with a high BMI had an increased PWV_CF_ and AI after adjustment for other CVD risk factors and other confounders such as age, HR, height, FBG, SBP, TC, smoker, and ethnicity [27]. Subjects in the current study were presented with various factors related to the CVD risk factors, thus, it was important to adjust for these factors. Previous studies found that the level of the pulse wave velocity (PWV_CF_) was increased among overweight and obese subjects [34] when compared to normal weight subjects. The augmentation index was found to be increased among overweight and obese Australian women [35]. A significant association between the fat mass and PWV among obese subjects and a significant association between the BMI and PWV in middle-aged adults had been observed in previous studies [36,37]. Obese children and adolescents with the metabolic syndrome (MS) had a higher PWV compared to obese subjects with no MS [38]. The ambulatory arterial stiffness index (AASI) was significantly higher in obese children compared to the controls [39].

However, our findings were not aligned with a few previous studies. No difference in the AI and PWV_CF_ was found among obese premenopausal women in the study by Ounis-Skali et al. (2007) [40]. No difference for the aortic PWV was found among overweight and obese young men in the study by Kappus et al. (2013) [41]. The discrepancy may be due to differences in age and sex.

### 4.2. Vascular Dysfunction in Obesity: Low Testosterone and Increased Inflammation

In this study, the high BMI was associated with a lower testosterone level. This was supported by previous studies [8,42,43]. The relationship is well established and appears to be bidirectional [8]. The lower testosterone level was associated with the decreased vascular function in this study whereby the AI was associated with a low free testosterone level. A study by Corrigan III et al. (2015) among 237 healthy men aged 50 ± 12 years showed that low testosterone was associated with microvascular dysfunction as evidenced by the decreased digital reactive hyperemia index (RHI) and increased wave reflection or AI [15]. In 455 men with no significant cardiovascular disease, the aortic stiffness (by measuring PWV_CF_) was inversely correlated to the total testosterone after adjustment for the confounders (β = −0.365, *p* < 0.001) [14]. Treatment with testosterone among old people with hypogonadism showed an improvement in the vascular function [44,45]. A study by Groti et al. (2018) among obese hypogonadal diabetic men showed that the treatment with testosterone undecanoate for a year improved the flow-mediated dilation (FMD) significantly by 2.40% ± 4.16% when compared to the subjects that received the placebo [44]. Another study found that the treatment with testosterone for three to six months improved the RHI and AI among the hypogonadal subjects with a total testosterone of <350 ng/dL [45].

Several mechanisms had been implicated in relation to the testosterone and increased vascular function. In vitro and in vivo experimental studies showed that testosterone caused a vasorelaxation through several mechanisms such as endothelium-independent pathways, the opening of the potassium channel or calcium antagonistic effects [46]. The testosterone also increased endothelial progenitor cells, lowered asymmetrical dimethylarginine (ADMA) levels and reduced the oxidative stress [47,48,49]. In this study, the total and free testosterone was correlated with the hs-CRP. A previous study found that the low testosterone was also associated with an increased inflammation [50], which was found to be associated with low testosterone levels among CAD, DM, and hypogonadal patients [51,52,53,54,55]. The anti-inflammatory effects of testosterone were demonstrated when it reduced the expression and secretion of TNF-α and IL-1β in monocyte-derived macrophages obtained from patients with coronary heart disease [56]. Another study found that the testosterone treatment led to the suppression of TNF, IL-1, and IL-6 released from cultured peripheral blood monocytes isolated from type 2 diabetes mellitus men with an androgen deficiency [57].

The vascular dysfunction among obese subjects may also be due to an increased inflammation. In this study, both the PWV_CF_ and AI were associated with the hs-CRP. The increased inflammation may lead to the increased arterial stiffness by several mechanisms, which has been extensively reviewed by previous authors [58,59]. Inflammation leads to the dysfunction of the endothelium, migration of the smooth muscle cell, calcification of the vascular wall, elastolysis, increased oxidative stress, high activity of metalloproteinases, degradation of collagen and extracellular matrix, and formation of uncoiled, stiffer collagen [58]. Lifestyle modifications such as aerobic exercise and dietary modification among obese subjects may reduce the arterial stiffness associated with a reduction in inflammatory markers such as IL-6 [60].

The synergistic effect of testosterone and hs-CRP on the vascular function had not been investigated previously. It was found that the combination of low FT and increased hs-CRP were associated with a higher augmentation index when compared to those with only an increased hs-CRP but high FT. This signifies that both factors may have synergistic effects that contributed to the vascular dysfunction and may have a different mechanism independent of each other as stated above. The presence of both factors, the low FT and high hs-CRP, may lead to a higher risk of vascular damage. In this study, FT correlated with the AI and not the TT. This may be due to the fact that FT is the fraction of testosterone that is free to act on cellular targets. TT is all of the testosterone in the blood, including those bound with the sex hormone binding globulin (SHBG) and albumin, which may not be free to act on biological targets. Thus, despite being a convenient clinical measure of the androgenic status, TT may not be the most reliable indicator to predict biological activities of testosterone in the body [61].

Several limitations need to be considered for the interpretation of the data in this study. Firstly, this was a cross-sectional study so causality between vascular functions and variables of interest cannot be firmly established. Secondly, the subjects consisted of Malays and Chinese only, which did not truly represent the multiracial Malaysian population. Thirdly, the level of obesity was determined by the BMI, which was assumed to be correlated with the level of adiposity. However, the level of adiposity is more accurately assessed by the dual-energy x-Ray absorptiometry (DXA) or leptin-adjusted BMI [62]. These two data could not be produced since we were not measuring the DXA or blood leptin. Fourthly, the population involved subjects with diabetes, dyslipidemia, and hypertension, who were on medications and this may affect the results of vascular functions. The involvement of those with CVD risk factors could not be avoided since this study was part of the metabolic syndrome study [27]. Lastly, the measurement of the BP was performed at one occasion and might not represent the true BP.

A further longitudinal study should be conducted to verify the associations between vascular functions, testosterone, and inflammation. An interventional study using a testosterone supplementation, anti-inflammation, and weight lost program may be beneficial to determine their effects towards the vascular function in the obese subjects [63].

## 5. Conclusions

Men with a high BMI suffer from an impaired vascular function, which is related to the low testosterone level and increased inflammation. The low testosterone level and increased inflammation may be the main mechanism in inducing the impaired vascular function among obese men. Weight loss, testosterone supplementation and anti-inflammatory agents may be beneficial for men to prevent the vascular dysfunction.

## Figures and Tables

**Table 1:** Biophysical and biochemical profiles of the subjects.

Characteristic	All (n = 303)	NG (n = 154)	OG (n = 149)	*p*
Age (years)	54.46 ± 9.77	54.46 ± 10.41	54.39 ± 9.23	0.43
BMI (kg/m^2^)	25.45 ± 4.26	22.20 ± 1.94	28.87 ± 3.24	<0.01
WC (cm)	98.10 ± 9.48	92.08 ± 6.96	104.22 ± 7.72	<0.01
SBP (mmHg)	141.78 ± 20.12	138.01 ± 18.41	144.92 ± 19.96	<0.01
DBP (mmHg)	85.64 ± 11.08	83.29 ± 9.84	87.94 ± 11.64	<0.01
HR (bpm)	61.17 ± 11.08	58.84 ± 9.07	63.23 ± 10.54	<0.01
PWV_CF_ (m/s)	8.54 ± 1.38	8.52 ± 1.42	8.55 ± 1.34	0.88
				0.02 *
AI (%)	16.33 ± 5.74	15.88 ± 5.58	16.91 ± 6.00	0.12
				<0.01 *
TC (mmol/L)	5.67 ± 1.84	5.70 ± 0.94	5.66 ± 1.14	0.70
FBG (mmol/L)	6.18 ± 1.05	5.70 ± 1.10	6.64 ± 2.25	<0.01
TG (mmol/L)	1.75 ± 1.49	1.52 ± 1.14	2.09 ± 1.85	<0.01
HDL (mmol/L)	1.23 ± 0.26	1.31 ± 0.26	1.16 ± 0.23	<0.01
LDL (mmol/L)	3.62 ± 0.91	3.66 ± 0.83	3.57 ± 0.94	0.40
TT (nmol/L)	18.72 ± 6.76	21.13 ± 6.44	16.18 ± 6.16	<0.01 **
FT (nmol/L)	0.37 ± 0.12	0.39 ± 0.11	0.34 ± 0.12	<0.01 **
Hs-CRP (mmol/L)	0.70 (2.08)	0.50 (1.5)	1.05 (2.8)	0.01
HPT (%)	53.2	47.8	58.7	0.09
DM (%)	14.1	6.8	21.7	<0.01
Dyslipidaemia (%)	74.3	72.3	76.4	0.40
Smoker (%)	24.3	21.1	27.7	0.19

Data are a mean ± SD except for high sensitivity C-reactive protein (hs-CRP), which is the median (interquartile range). * After adjustments for other cardiovascular disease (CVD) risk factors (age, heart rate (HR), height, fasting blood glucose (FBG), systolic blood pressure (SBP), total cholesterol (TC), smoker, race), ** After adjustment for age and race.

**Table 2:** The associations between the total testosterone (TT), free testosterone (FT) and high sensitivity C-reactive protein (hs-CRP) with other cardiovascular disease (CVD) risk factors and vascular functions.

	TT		FT		Hs-CRP	
	*R*	*p*	*R*	*p*	rho	*p*
Age (years)	−0.11	<0.05	−0.30	<0.01	0.04	0.50
WC (cm)	−0.38	<0.01	−0.22	<0.01	0.24	<0.01
BMI (kg/m^2^)	−0.45	<0.01	−0.30	<0.01	0.26	<0.01
SBP (mmHg)	−0.14	0.01	−0.18	<0.01	0.16	<0.01
DBP (mmHg)	−0.10	0.08	−0.10	0.09	0.19	<0.01
PWV_CF_ (m/s)	0.01	0.92	0.01	0.92	0.11	<0.05
AI (%)	−0.06	0.32	−0.13	<0.05	0.14	0.01
TC (mmol/L)	−0.02	0.72	−0.04	0.44	0.12	<0.05
TG (mmol/L)	−0.04	0.45	0.02	0.69	0.10	0.08
HDL (mmol/L)	0.11	0.07	0.01	0.81	−0.19	<0.01
LDL (mmol/L)	0.01	0.95	0.04	0.48	0.14	<0.05
Hs-CRP (mg/L)	−0.16	<0.01	−0.13	<0.05	-	-

**Table 3:** Subjects characteristics and vascular functions according to the group of the high and low level of FT and hs-CRP.

	G1 (n = 78)	G2 (n = 70)	G3 (n = 75)	G4 (n = 80)	*p*
Age (years)	51.46 ± 9.83	51.44 ± 8.25	58.33 ± 9.87	56.90 ± 9.34	<0.01
WC (cm)	93.70 ± 8.28	97.87 ± 7.31	98.10 ± 7.51	101.94 ± 11.74	<0.01
BMI (kg/m^2^)	23.38 ± 3.21	25.00 ± 3.36	25.59 ± 4.00	27.66 ± 4.76	<0.01
SBP (mmHg)	136.13 ± 19.06	138.64 ± 17.82	142.47 ± 18.09	149.30 ± 20.62	<0.01
DBP (mmHg)	82.99 ± 10.47	85.32 ± 9.80	85.13 ± 11.64	89.36 ± 11.63	<0.01
HR (bpm)	58.60 ± 8.87	62.28 ± 11.50	60.65 ± 10.56	62.65 ± 9.20	0.05
FBG (mmol/L)	5.93 ± 1.47	5.95 ± 1.75	6.14 ± 1.70	6.63 ± 2.30	0.10
TC (mmol/L)	5.45 ± 0.85	5.68 ± 1.12	5.69 ± 1.01	5.92 ± 1.13	<0.05
TG (mmol/L)	1.35 ± 1.01	2.08 ± 2.30	1.84 ± 1.21	1.95 ± 1.39	<0.05
HDL (mmol/L)	1.31 ± 0.26	1.18 ± 0.27	1.25 ± 0.23	1.19 ± 0.25	<0.05
LDL (mmol/L)	3.46 ± 0.78	3.61 ± 0.92	3.64 ± 0.91	3.78 ± 0.94	0.16
Hs-CRP (mg/L)	0.30 (0.40)	2.05 (2.45)	0.30 (0.30)	2.95 (5.10)	<0.01
FT (nmol/L)	0.46 ± 0.07	0.46 ± 0.08	0.28 ± 0.07	0.27 ± 0.07	<0.01
PWV_CF_ (m/s)	8.28 ± 1.35	8.54 ± 1.52	8.60 ± 1.33	8.78 ± 1.32	0.15
AI (%)	14.65 ± 5.37	15.80 ± 5.51	16.65 ± 5.35	18.33 ± 6.43	<0.01

Data are presented as a mean ± SD except for the hs-CRP and median (IQR). G1 = high FT and low hs-CRP, G2 = high FT and high hs-CRP, G3 = low FT and low CRP, and G4 = low FT and high CRP.

## Abbreviations

AASI	Ambulatory arterial stiffness index
AI	Augmentation index
BMI	Body mas index
BP	Blood pressure
CRP	C-reactive protein
CVD	Cardiovascular disease
DBP	Diastolic blood pressure
DM	Diabetes mellitus
DPW	Distance of the pressure wave travel
DXA	Duel-energy x-Ray absorptiometry
FBG	Fasting blood glucose
FT	Free testosterone
hCG	Human chorionic gonadotrophin
HDL	High density lipoprotein
HPT	Hypertension
HR	Heart rate
Hs-CRP	High sensitivity C-reactive protein
IL	Interleukin
LDL	Low density lipoprotein
LH	Luteinising hormone
MS	Metabolic syndrome
NG	Low/normal body mass index group
OG	High BMI group
PWV_CF_	Carotid femoral pulse wave velocity
RHI	Reactive hyperemia index
SBP	Systolic blood pressure
SHBG	Sex hormone binding globulin
TC	Total cholesterol
TG	Triglyceride
TNF-α	Tumour necrosis factor-alpha
TT	Total testosterone
Tt	Transit time
WC	Waist circumference

## References

[1] Jaacks L.M., Vandevijvere S., Pan A., McGowan C.J., Wallace C., Imamura F., Mozaffarian D., Swinburn B., Ezzati M. (2019). The obesity transition: Stages of the global epidemic. Lancet Diabetes Endocrinol..

[2] Fatimah S., Tahir A., Siti Sa’adiah H.N., Maimunah A.H. (1999). Nutritional Status of Adults Aged 18 Years and above. National Health and Morbidity Survey 1996.

[3] Institute for Public Health (IPH) (2015). National Health and Morbidity Survey 2015 (NHMS 2015). Vol. II: Non-Communicable Diseases, Risk Factors & Other Health Problems.

[4] Jia G., Aroor A.R., DeMarco V.G., Martinez-Lemus L., Meininger G.A., Sowers J.R. (2015). Vascular stiffness in insulin resistance and obesity. Front. Physiol..

[5] Avolio A.P., Kuznetsova T., Heyndrickx G.R., Kerkhof P.L.M., Li J.K.J., Kerkhof P., Miller V. (2018). Arterial Flow, Pulse Pressure and Pulse Wave Velocity in Men and Women at Various Ages. Sex-Specific Analysis of Cardiovascular Function.

[6] Ben-Shlomo Y., Spears M., Boustred C., May M., Anderson S.G., Benjamin E.J., Boutouyrie P., Cameron J., Chen C.H., Cruickshank J.K. (2014). Aortic pulse wave velocity improves cardiovascular event prediction: An individual participant meta-analysis of prospective observational data from 17,635 subjects. J. Am. Coll. Cardiol..

[7] Chirinos J.A., Kips J.G., Jacobs D.R., Brumback L., Duprez D.A., Kronmal R., Bluemke D.A., Townsend R.R., Vermeersch S., Segers P. (2012). Arterial wave reflections and incident cardiovascular events and heart failure: MESA (Multiethnic Study of Atherosclerosis). J. Am. Coll. Cardiol..

[8] Kelly D.M., Jones T.H. (2015). Testosterone and obesity. Obes. Rev..

[9] Saboor Aftab S.A., Kumar S., Barber T.M. (2013). The role of obesity and type 2 diabetes mellitus in the development of male obesity-associated secondary hypogonadism. Clin. Endocrinol..

[10] Dandona P., Dhindsa S. (2011). Update: Hypogonadotropic hypogonadism in type 2 diabetes and obesity. J. Clin. Endocrinol. Metab..

[11] Pitteloud N., Dwyer A.A., DeCruz S., Lee H., Boepple P.A., Crowley W.F., Hayes F.J. (2008). The relative role of gonadal sex steroids and gonadotropin-releasing hormone pulse frequency in the regulation of follicle-stimulating hormone secretion in men. J. Clin. Endocrinol. Metab..

[12] Hamilton E., Gianatti E., Strauss B., Wentworth J., Lim-Joon D., Bolton D., Zajac J.D., Grossmann M. (2011). Increase in visceral and subcutaneous abdominal fat in men with prostate cancer treated with androgen deprivation therapy. Clin. Endocrinol. (Oxf.).

[13] Isidori A.M., Caprio M., Strollo F., Moretti C., Frajese G., Isidori A., Fabbri A. (1999). Leptin and androgens in male obesity: Evidence for leptin contribution to reduced androgen levels. J. Clin. Endocrinol. Metab..

[14] Vlachopoulos C., Ioakeimidis N., Miner M., Aggelis A., Pietri P., Terentes-Printzios D., Tsekoura D., Stefanadis C. (2014). Testosterone deficiency: A determinant of aortic stiffness in men. Atherosclerosis.

[15] Corrigan F.E., Al Mheid I., Eapen D.J., Hayek S.S., Sher S., Martin G.S., Quyyumi A.A. (2015). Low testosterone in men predicts impaired arterial elasticity and microvascular function. Int. J. Cardiol..

[16] Lafontan M. (2004). Fat cells: Afferent and efferent messages define new approaches to treat obesity. Ann. Rev. Pharmacol. Toxicol..

[17] Cinti S., Mitchell G., Barbatelli G., Murano I., Ceresi E., Faloia E., Wang S., Fortier M., Greenberg A.S., Obin M.S. (2005). Adipocyte death defines macrophage localization and function in adipose tissue of obese mice and humans. J. Lipid Res..

[18] Karastergiou K., Mohamed-Ali V. (2010). The autocrine and paracrine roles of adipokines. Mol. Cell. Endocrinol..

[19] Ellulu M.S., Khaza’ai H., Abed Y., Rahmat A., Ismail P., Ranneh Y. (2015). Role of fish oil in human health and possible mechanism to reduce the inflammation. Inflammopharmacology.

[20] Daiber A., Steven S., Weber A., Shuvaev V.V., Muzykantov V.R., Laher I., Li H., Lamas S., Münzel T. (2017). Targeting vascular (endothelial) dysfunction. Br. J. Pharmacol..

[21] Chin K.Y., Wan Ngah W.Z., Ima-Nirwana S. (2016). Lessons from the Bone Chapter of the Malaysian Aging Men Study. Int. J. Environ. Res. Public Health.

[22] Aminuddin A., Chellappan K., Maskon O., Zakaria Z., Karim A.A., Ngah W.Z., Nordin N.A.M. (2014). Augmentation index is a better marker for cardiovascular risk in young Malaysian males. A comparison of involvement of pulse wave velocity, augmentation index, and C-reactive protein. Saudi Med. J..

[23] Alberti K.G., Zimmet P.Z. (1998). Definition, diagnosis and classification of diabetes mellitus and its complications. Part 1: Diagnosis and classification of diabetes mellitus provisional report of a WHO consultation. Diabet. Med..

[24] Chobanian A.V., Bakris G.L., Black H.R., Cushman W.C., Green L.A., Izzo J.L., Jones D.W., Materson B.J., Oparil S., Wright J.T. (2003). The seventh report of the joint national committee on prevention, detection, evaluation, and treatment of high blood pressure: The JNC 7 report. Jama.

[25] Stone N.J., Bilek S., Rosenbaum S. (2005). Recent National Cholesterol Education Program Adult Treatment Panel III update: Adjustments and option. Am. J. Cardiol..

[26] WHO (1998). Obesity: Preventing and Managing the Global Epidemic. Proceedings of the Report on a WHO Consultation on Obesity.

[27] Aminuddin A., Zakaria Z., Fuad A.F., Kamsiah J., Othman F., Das S., Kamisah Y., Qodriyah H.S., Jubri Z., Nordin N.A.M. (2013). High C reactive protein associated with increased pulse wave velocity among urban men with metabolic syndrome in Malaysia. Saudi Med. J..

[28] Laurent S., Cockcroft J., Bortel L.V., Boutouyrie P., Giannattasio C., Hayoz D., Pannier B., Vlachopoulos C., Wilkinson I., Struijker-Boudier H. (2007). Abridged version of the expert consensus document on arterial stiffness. Artery Res..

[29] Chin K.Y., Soelaiman I.N., Mohamed I.N., Ngah W.Z. (2012). Serum testosterone, sex hormone-binding globulin and total calcium levels predict the calcaneal speed of sound in men. Clinics (Sao Paulo).

[30] Sodergard R., Backstrom T., Shanbhag V., Carstensen H. (1982). Calculation of free and bound fractions of testosterone and estradiol-17 beta to human plasma proteins at body temperature. J. Steroid Biochem..

[31] Tan C.E., Ma S., Wai D., Chew S.K., Tai E.S. (2004). Can we apply the National Cholesterol Education Program Adult Treatment Panel definition of the metabolic syndrome to Asians?. Diabetes Care.

[32] Deurenberg-Yap M., Chew S.K., Deurenberg P. (2002). Elevated body fat percentage and cardiovascular risks at low body mass index levels among Singaporean Chinese, Malays and Indians. Obes. Rev..

[33] Deurenberg P., Deurenberg-Yap M., Guricci S. (2002). Asians are different from Caucasians and from each other in their body mass index/body fat percent relationship. Obes. Rev..

[34] Miyaki A., Maeda S., Choi Y., Akazawa N., Eto M., Tanaka K., Ajisaka R. (2013). Association of plasma pentraxin 3 with arterial stiffness in overweight and obese individuals. Am. J. Hypertens..

[35] Pal S., Radavelli-Bagatini S. (2013). Association of arterial stiffness with obesity in Australian women: A pilot study. J. Clin. Hypertens..

[36] Arner P., Bäckdahl J., Hemmingsson P., Stenvinkel P., Eriksson-Hogling D., Näslund E., Thorell A., Andersson D.P., Caidahl K., Rydén M. (2015). Regional variations in the relationship between arterial stiffness and adipocyte volume or number in obese subjects. Int. J. Obes..

[37] Strasser B., Arvandi M., Pasha E.P., Haley A.P., Stanforth P., Tanaka H. (2015). Abdominal obesity is associated with arterial stiffness in middle-aged adults. Nutr. Metab. Cardiovasc. Dis..

[38] Manco M., Nobili V., Alisi A., Panera N., Handberg A. (2017). Arterial stiffness, thickness and association to suitable novel markers of risk at the origin of cardiovascular disease in obese children. Int. Res. Med. Sci..

[39] Saner C., Simonetti G.D., Wühl E., Mullis P.E., Janner M. (2015). Increased ambulatory arterial stiffness index in obese children. Atherosclerosis.

[40] Ounis–Skali N., Bentley–Lewis R., Mitchell G.F., Solomon S., Seely E.W. (2007). Central aortic pulsatile hemodynamics in obese premenopausal women. J. Am. Soc. Hypertens..

[41] Kappus R.M., Fahs C.A., Smith D., Horn G.P., Agiovlasitis S., Rossow L., Jae S.Y., Heffernan K.S., Fernhall B. (2013). Obesity and overweight associated with increased carotid diameter and decreased arterial function in young otherwise healthy men. Am. J. Hypertens..

[42] Bekaert M., Van Nieuwenhove Y., Calders P., Cuvelier C.A., Batens A.H., Kaufman J.M., Ouwens D.M., Ruige J.B. (2015). Determinants of testosterone levels in human male obesity. Endocrine.

[43] Khaw K.T., Barrett-Connor E. (1992). Lower endogenous androgens predict central adiposity in men. Ann. Epidemiol..

[44] Groti K., Žuran I., Antonič B., Foršnarič L., Pfeifer M. (2018). The impact of testosterone replacement therapy on glycemic control, vascular function, and components of the metabolic syndrome in obese hypogonadal men with type 2 diabetes. Aging Male.

[45] Shoskes D.A., Tucky B., Polackwich A.S. (2016). Improvement of endothelial function following initiation of testosterone replacement therapy. Transl. Androl. Urol..

[46] Lopes R.A.M., Neves K.B., Carneiro F.S., Tostes R. (2012). Testosterone and vascular function in aging. Front. Physiol..

[47] Foresta C., Zuccarello D., DeToni L., Garolla A., Caretta N., Ferlin A. (2008). Androgens stimulate endothelial progenitor cells through an androgen receptor mediated pathway. Clin. Endocrinol. (Oxf.).

[48] Tsikas D., Kinzel M. (2018). Associations between asymmetric dimethylarginine (ADMA), nitrite-dependent renal carbonic anhydrase activity, and plasma testosterone levels in hypogonadal men. Hell. J. Cardiol..

[49] Hwang T.I., Liao T.L., Lin J.F., Lin Y.C., Lee S.Y., Lai Y.C., Kao S.H. (2011). Low-dose testosterone treatment decreases oxidative damage in TM3 Leydig cells. Asian J. Androl..

[50] Kelly D.M., Jones T.H. (2013). Testosterone: A vascular hormone in health and disease. J. Endocrinol..

[51] Yang Y.M., Lv X.Y., Huang W.D., Xu Z.R., Wu L.J. (2005). Study of androgen and Atherosclerosis in old-age male. J. Zhejiang Univ. Sci. B.

[52] Maggio M., Basaria S., Ble A., Lauretani F., Bandinelli S., Ceda G.P., Valenti G., Ling S.M., Ferrucci L. (2006). Correlation between testosterone and the inflammatory marker soluble interleukin-6 receptor in older men. J. Clin. Endocrinol. Metab..

[53] Kapoor D., Clarke S., Channer K.S., Jones T.H. (2007). The effect of testosterone replacement therapy on adipocytokines and C-reactive protein in hypogonadal men with type 2 diabetes. Eur. J. Endocrinol..

[54] Nettleship J.E., Pugh P.J., Channer K.S., Jones T., Jones R.D. (2007). Inverse relationship between serum levels of interleukin-1b and testosterone in men with stable coronary artery disease. Horm. Metab. Res..

[55] Pantea-Stoian A., Pituru S.M., Hainarosie R., Andronache L.F., Ginghina O., Serafinceanu C. (2018). Testosterone therapy, new opportunities in diabetes mellitus. Farmacia.

[56] Corcoran M.P., Meydani M., Lichtenstein A.H., Schaefer E.J., Dillard A., Lamon-Flava S. (2010). Sex hormone modulation of proinflammatory cytokine and C-reactive protein expression in macrophages from older men and postmenopausal women. J. Endocrinol..

[57] Corrales J.J., Almeida M., Burgo R., Mories M.T., Miralles J.M., Orfao A. (2006). Androgen-replacement therapy depresses the ex vivo production of inflammatory cytokines by circulating antigen-presenting cells in aging type-2 diabetic men with partial androgen deficiency. J. Endocrinol..

[58] Mozos I., Malainer C., Horbańczuk J., Gug C., Stoian D., Luca C.T., Atanasov A.G. (2017). Inflammatory markers for arterial stiffness in cardiovascular diseases. Front. Immunol..

[59] Lyle A.N., Raaz U. (2017). Killing me unsoftly: Causes and mechanisms of arterial stiffness. Arterioscler. Thromb. Vasc. Biol..

[60] Kumagai H., Zempo-Miyaki A., Yoshikawa T., Eto M., So R., Tsujimoto T., Nishiyasu T., Tanaka K., Maeda S. (2017). Which cytokine is the most related to weight loss-induced decrease in arterial stiffness in overweight and obese men?. Endocr. J..

[61] Krakowsky Y., Grober E.D. (2015). Testosterone Deficiency-Establishing A Biochemical Diagnosis. EJIFCC.

[62] Shah N.R., Braverman E.R. (2012). Measuring adiposity in patients: The utility of body mass index (BMI), percent body fat, and leptin. PLoS ONE.

[63] Grossmann M. (2018). Hypogonadism and male obesity: Focus on unresolved questions. Clin. Endocrinol..

